# First person – Christy Tulen and Ying Wang

**DOI:** 10.1242/dmm.049477

**Published:** 2022-03-28

## Abstract

First Person is a series of interviews with the first authors of a selection of papers published in Disease Models & Mechanisms, helping early-career researchers promote themselves alongside their papers. Christy Tulen and Ying Wang are co-first authors on ‘ Dysregulated mitochondrial metabolism upon cigarette smoke exposure in various human bronchial epithelial cell models’, published in DMM. Christy is a PhD student in the lab of Prof. Frederik-Jan van Schooten at Maastricht University Medical Center+, Maastricht, The Netherlands, investigating the mechanistic involvement of smoking-associated aldehyde-induced mitochondrial dysfunction in chronic obstructive pulmonary disease lung pathology. Ying is a PhD student in the lab of Prof. Pieter S. Hiemstra at Leiden University Medical Center, Leiden, The Netherlands, investigating the interaction between respiratory viruses (SARS-CoV-2 and rhinovirus) and human lung epithelial cells.

## How would you explain the main findings of your paper to non-scientific family and friends?

**CT:** Tobacco smoking is known to be an important risk factor in the development of chronic obstructive lung disease (COPD), but the molecular mechanisms playing a role in the pathogenesis are incompletely understood. As it is important to understand the molecular pathways involved in this lung disease to develop future potential therapies, we aim to investigate the impact of smoking on the molecular regulation of important molecules involved in the regulation of the ‘energy fabrics’ of the lung cells called mitochondria. To study this, we cultured airway epithelial cells from different ‘healthy’ human donors in a dish including undifferentiated basal cells or at the air–liquid interface, a differentiated multi-cellular lung model including cells that are responsible for the production of mucus and formation of cilia. These various lung cell models were exposed to the smoke of a research cigarette or cigarette smoke extract (i.e. smoke bubbled through liquid), once or multiple times during differentiation, in order to investigate the impact of smoke exposure on the molecular regulation of the mitochondria in an intact cell layer, a damaged epithelial layer or repair mechanism. We observed that smoke exposure affected the abundance of molecules involved in the regulation of the breakdown of mitochondria in all models, while exposure to smoke differentially affected the regulation of molecules related to the generation of mitochondria. Conclusively, these results highlight the importance of tailoring the experimental model to the research question.

**Figure:**
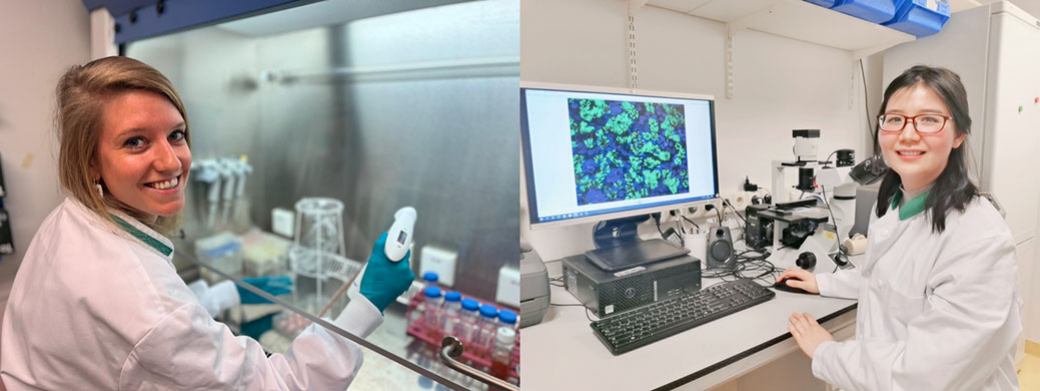
Christy Tulen (left) and Ying Wang (right)

**Figure:**
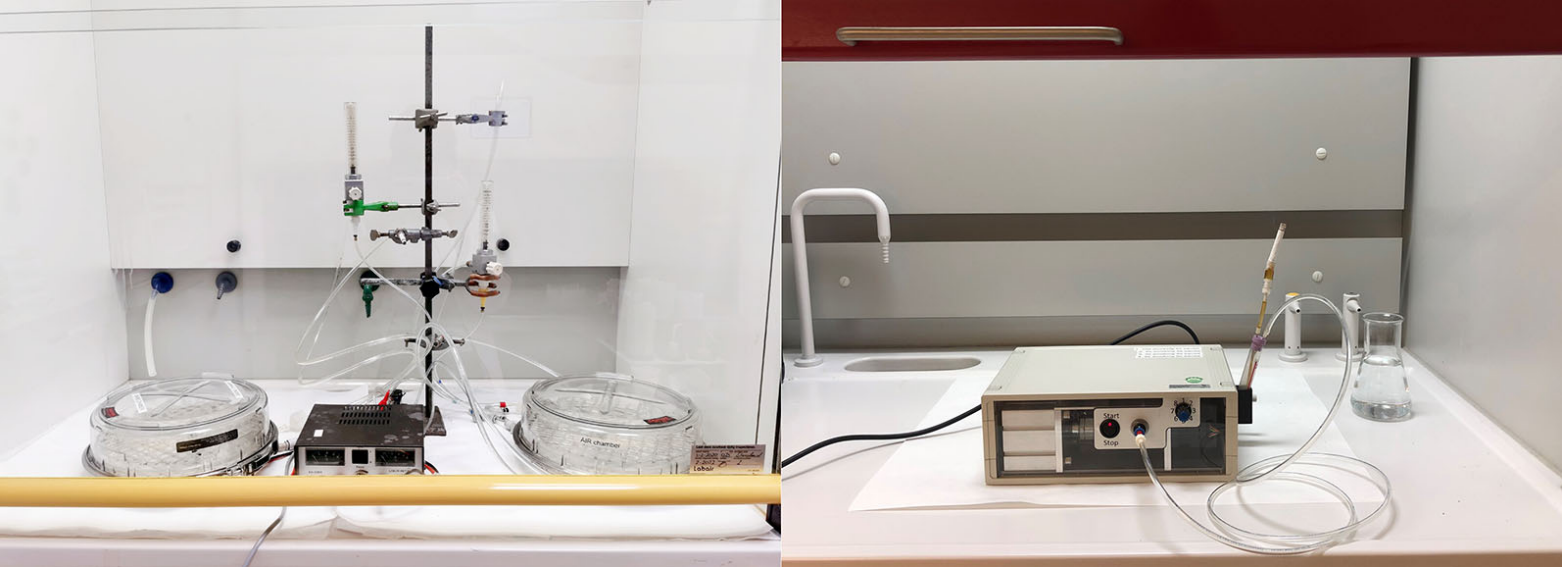
**Representation of the various smoke exposure models used in our study.** Whole cigarette smoke was generated (left) to expose human primary bronchial epithelial cells in undifferentiated and differentiated cultures, and cigarette smoke extract was generated (right) to expose undifferentiated human primary bronchial epithelial cells.

**YW:** Smoking, a big threat for human health, causes many chronic diseases, including COPD. This kind of disease generally induces breathing difficulties and limits people's behaviour during normal life. Therefore, it is crucial to study how smoking damages the respiratory tract using cells isolated from human lung. To mimic smoking, cells cultured on a plate were exposed to whole cigarette smoke from a research cigarette in a chamber or cigarette smoke extract was added directly to cultures. Possible changes were investigated using multiple technologies.

“Our study shows the robust model-independent impact of smoke exposure on the abundance of key molecules involved in autophagy and receptor-mediated mitophagy.”

## What are the potential implications of these results for your field of research?

**CT/YW:** Although previous clinical, animal *in vivo* and *in vitro* studies have shown that smoking modulates mitochondrial function, and recent studies suggested a critical role for mitochondrial dysfunction in the pathogenesis of smoke-related lung diseases such as COPD, the impact of smoke exposure on the molecular mechanisms involved in mitochondrial quality control in airway epithelial cells needs to be stressed using advanced *in vitro* models. In our study, we compared various smoke-exposure cell models ranging from cigarette smoke extract-treated undifferentiated human primary bronchial epithelial cells (PBEC) to well-differentiated PBEC cultures exposed to whole cigarette smoke, respectively, acutely or chronically followed by smoking cessation. Our study shows the robust model-independent impact of smoke exposure on the abundance of key molecules involved in autophagy and receptor-mediated mitophagy. With our study, we aim to contribute to the insights into the molecular mechanisms, in particular related to mitochondrial metabolism, driving smoke-induced COPD pathogeneses. Moreover, our study highlights the importance of making considered choices for (un)differentiated cell models, and type and duration of smoke exposure in future respiratory inhalation (toxicology) studies tailored to the research question. For example, more advanced models including air–liquid interface-differentiated PBEC models in combination with whole cigarette smoke exposure are reflecting different parts of the airway epithelium and types of smoke exposure compared to the simple conventional submerged undifferentiated PBEC models exposed to cigarette smoke extract.

## What are the main advantages and drawbacks of the model system you have used as it relates to the disease you are investigating?

**CT/YW:** The main advantage of our model system used is studying, for the first time, a comprehensive panel of markers involved in molecular processes associated with the regulation of mitochondrial metabolism in human PBEC from various donors cultured in both (un)differentiated submerged and air–liquid interface conditions, as well as the comparison of different type and duration of cigarette smoke exposure. Previous studies often focus on either studying a selection of mitochondrial markers or using only the simpler submerged (cell-line) culture models. Using these relevant *in vitro* models mimicking the pseudostratified epithelium (differentiated PBEC) or the damaged epithelium layer consisting of basal cells (undifferentiated PBEC) in combination with various smoke exposure regimes made it possible to investigate the impact of smoke on the regulation of mitochondrial metabolism in various stages of COPD, an intact cell layer, damaged epithelial layer or capability of repair mechanism. Our findings highlighted the robust effects of smoking on the abundance of regulators involved in mitochondrial quality control, but also addressed differences in the regulation of mitochondrial metabolism among different cell models.

A drawback of our study is that our model system is limited to airway bronchial epithelial cells, resulting in a lack of communication with other cells, like immune cells. More complex *in vitro* models are needed to imitate and study the impact of the cell–cell interaction in the lung, for example by using co-cultures of epithelial cells and monocyte-derived macrophages.

## What has surprised you the most while conducting your research?

**CT:** The development of a pseudostratified epithelium mimicking the human lung epithelium in a dish fascinated me from the beginning of my research. Starting up a culture with basal cells, which are capable of differentiation into mucus-producing cells and cilia, reflects the (regenerative) capacity of the human lung. It is interesting to study the different cell models reflecting different disease stages, an intact cell layer, damaged epithelial layer or capability of repair mechanism in this study. Moreover, it has been interesting to observe the inter-donor variability between the human PBEC from various donors in response to smoke exposure in our study. Using various cell models with PBEC of different donors, highlights the importance of choosing the right model tailoring the research question.

**YW:** When we performed our study, I was surprised at the various cell models. The difference among these models, including cell types, methods and duration of cigarette smoke exposure, affected the effects of smoking on the regulation of mitochondrial quality control processes in airway epithelial cells, which highlights the importance of choosing suitable cell models.

## Describe what you think is the most significant challenge impacting your research at this time and how will this be addressed over the next 10 years?

**CT:** Recent research is focusing on the development of advanced state-of-the art *in vitro* models representing the human lung for example 2D or 3D models, co-cultures, lung on a chip or *ex vivo* precision-cut lung slices. In the next 10 years, I expect that these models will be more representative, standardized, and cost and time efficient. Besides, with the development of those advanced and donor-specific cultures, more patient (group)-specific information about the molecular mechanisms underlying diseases will be elucidated, further contributing to the shift to personalized medicine. Moreover, focusing on these advanced *in vitro* models will result in the development of new research tools to study molecular pathways such as analysing mitochondrial function via respiratory analysis in those models, which is not possible yet in, for example, air–liquid interface PBEC cultures.

**YW:** A major but important challenge for the coming years is to link mitochondrial function to clinical symptoms. For instance, ‘cytokine storms’ are known to contribute to tissue damage and organ failure. However, there is no clear evidence so far to address if mitochondrial dysfunction is associated with inflammation and immune responses. Moreover, patients who suffer from the same mitochondrial disease can have differences in symptoms, severity and age of onset. In the next 10 years, I predict that elucidating the role of mitochondrial dysfunction in specific clinical symptoms will be possible following novel technologies like cytometry by time of flight (CYTOF) and single-cell RNA sequencing.

“[…] it is important for early-career scientists to collaborate with various labs and scientists, both nationally and internationally, within their field of research during their early career.”

## What changes do you think could improve the professional lives of early-career scientists?

**CT:** In my opinion, it is important for early-career scientists to collaborate with various labs and scientists, both nationally and internationally, within their field of research during their early career. In this manner, they are able to gain (lab) experience in their field of research, and to get insight into and different points of view on their research topic, as each lab has their own specialization and they could build a professional network for further career opportunities. Finally, yet importantly, working together could move research projects to the next level as together you are able to join forces.

**YW:** I think suggestions from experts and professional career sharing are very important. From the beginning of your project, it is important to evaluate your project set-up and results with professional experts, which will help to quickly modulate your research direction. Moreover, professional career sharing from people in business or academia will help to know the requirements of your ideal job in the future.

## What's next for you?

**CT/YW:** In terms of this paper, we have studied the effects of cigarette smoke exposure on the regulation of mitochondrial quality control and metabolism in multiple airway cell models, highlighting the importance of tailoring the model to the research question. As we are both working on unravelling the molecular mechanisms underlying the pathogenesis of COPD, we are focusing on different aspects during follow-up studies in our PhD project.

**CT:** In my project, we aim to investigate the impact of smoking-associated exposure of aldehydes on the molecular regulation of mitochondrial metabolism using *in vivo* and *in vitro* PBEC models. During the pyrolysis and combustion of tobacco, several aldehydes are generated including acetaldehyde, acrolein and formaldehyde. These reactive short-chain aldehydes induce cellular mechanisms underlying respiratory toxicity and are shortlisted for future regulation. Although impaired mitochondrial morphology and function have been described in cigarette smoke- and acrolein-exposed human airway epithelial cells, the effect of smoking-associated aldehydes exposure on the molecular pathways involved in mitochondrial content and mitochondrial quality control (mitochondrial biogenesis versus mitophagy) has to be elucidated.

**YW:** In my project, we aim to study if smoking-disrupted mitochondria play a pivotal role in respiratory virus infection. Previous researchers have identified that smoking can contribute to viral infection. For instance, it has been shown that COPD patients are more susceptible to viral infection, further accelerating severity of diseases. Recently, the COVID-19 pandemic also shows an association between smoking and SARS-CoV-2 infection. Altogether, mechanisms of smoke-induced viral replication need to be stressed in the future.
